# Chest Pain Resolution with His-bundle Pacing in a Patient with Left Bundle Branch Block–related Nonischemic Left Ventricular Dysfunction

**DOI:** 10.19102/icrm.2019.100906

**Published:** 2019-09-15

**Authors:** Martha G. Ferrara, Roger V. Cappucci, Daniel Y. Wang

**Affiliations:** ^1^White Plains Hospital Center, Montefiore Health System, White Plains, NY, USA

**Keywords:** Chest pain syndrome, His-bundle pacing, left bundle branch block, remote monitoring

## Abstract

Chest pain in patients with left bundle branch block (LBBB) and normal coronaries has been reported previously in the literature. Prior cases of intermittent LBBB and “chest pain syndrome” are known of, but the causes of and treatment options for such remain unclear. A mechanism of myocardial dyssynchrony has been proposed as a possible cause of the pain, but this has not yet widely been investigated. The application of His-bundle pacing techniques to promote normal activation of the conduction system may be a treatment option. The function of cardiac implantable electronic devices can be followed via remote monitoring (RM), a vital tool in this unique patient population. The present report introduces the case of a 51-year-old female to highlight this under-recognized syndrome, including the pacing technologies used for treatment and the crucial role of RM follow-up in such affected individuals.

## Introduction

Patients presenting with chest pain, left bundle branch block (LBBB), and normal coronaries have previously been discussed in the literature. However, though reports of intermittent LBBB and “chest pain syndrome” have been shared, the causes of and treatment options for such remain unclear. A mechanism of myocardial dyssynchrony has been proposed as a possible cause of the pain, but this possibility remains largely uninvestigated. The recent advent of His-bundle pacing techniques to promote normal activation of the conduction system represents a feasible treatment option. Cardiac implantable electronic devices (CIEDs) are followed via remote monitoring (RM), a vital tool in this unique patient population. This case study highlights this under-recognized syndrome, including the pacing technologies used for treatment and the crucial role of RM follow-up in these patients in order to inform health care professionals.

## Case presentation

**Figure 1:**
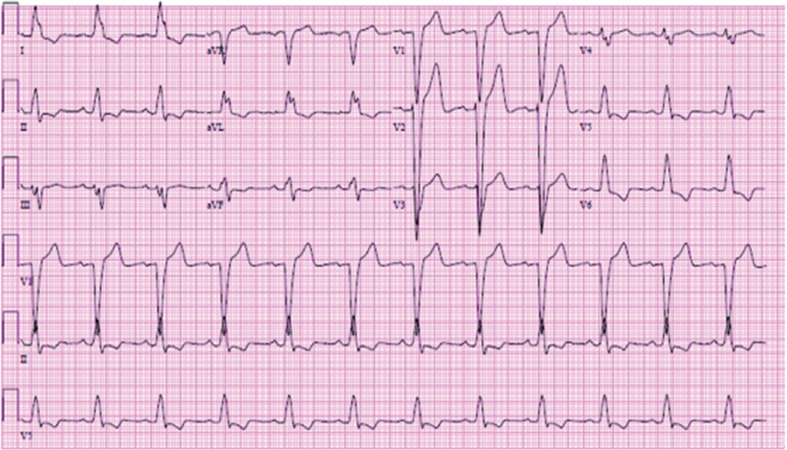
ECG showing left bundle branch block.

A 51-year old female with a history of hypertension, migraine, and nonradiating chest pain presented having visited the emergency room (ER) at least eight times previously for similar complaints. Her initial chest pain episode presentation was in November 2015, when an instance of left bundle branch block (LBBB) **(Figure 1)** was documented for the first time. Her echocardiogram results were normal, and cardiac catheterization showed no significant coronary artery disease. Subsequent electrocardiograms (ECGs) indicated LBBB resolution.

In October 2017, chest pain episodes prompted her to make subsequent ER visits with ECGs showing the return of LBBB. A repeat echocardiogram showed a reduced left ventricular (LV) ejection fraction (LVEF) of 40% to 45%. The results of a nuclear stress test were unremarkable.

**Figure 2:**
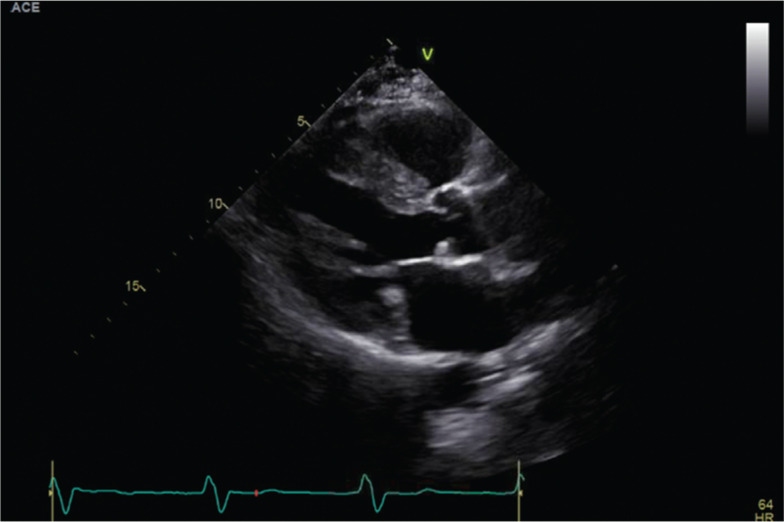
Echocardiogram before device implant.

In early March 2018, she was again admitted for chest pain; a repeat echocardiogram showed a LVEF of 54% with abnormal septal/anteroseptal wall motion consistent with conduction delay **(Figure 2)**. She was seen at this point by the electrophysiology (EP) service and was given an ambulatory cardiac telemetry monitor at discharge, which showed sinus rhythm with LBBB and intermittent 2:1 Mobitz II atrioventricular block.

**Figure 3:**
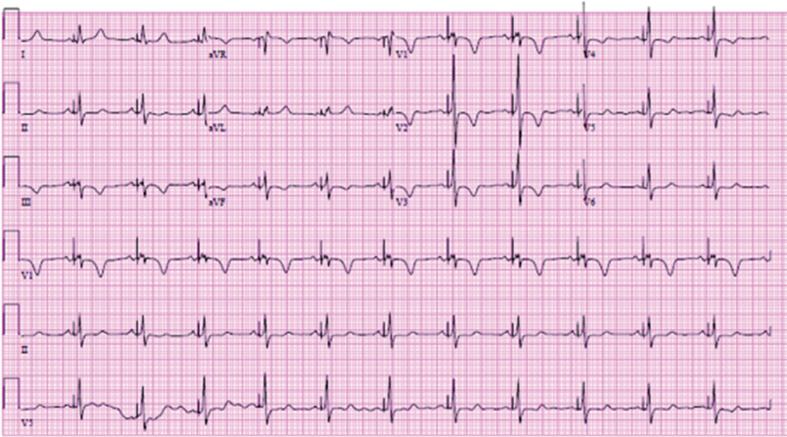
Initial ECG after device implant (April 2, 2018).

**Figure 4:**
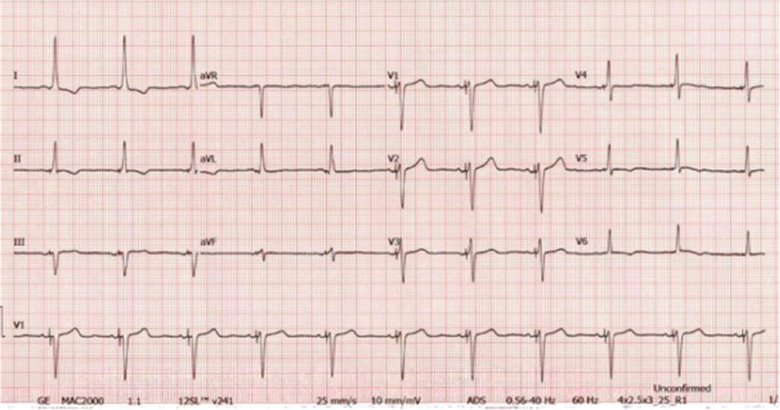
ECG with nonselective His-bundle pacing pattern.

In late March 2018, she presented to the ER with abrupt syncope and a head concussion. The patient underwent an EP study and, subsequently, a biventricular cardiac resynchronization therapy pacemaker (CRT-P) implantation including a His-bundle lead and a coronary sinus (CS) lead on April 2, 2018 **(Figure 3)**. His-bundle pacing resulted in LBBB resolution and narrow QRS complex, and her chest pain dissipated. RM services were instituted prior to discharge. Follow-up at one-month postimplantation showed checkup results, and the CS lead was programmed to an off status to allow the His-bundle lead to be the only pacing lead. The His-bundle lead checkup findings were similarly normal, with a threshold of 2.5 V at 1.0 ms. ECG revealed a nonselective His-bundle pacing pattern **(Figure 4)**; the patient reported no chest pain.

**Figure 5:**
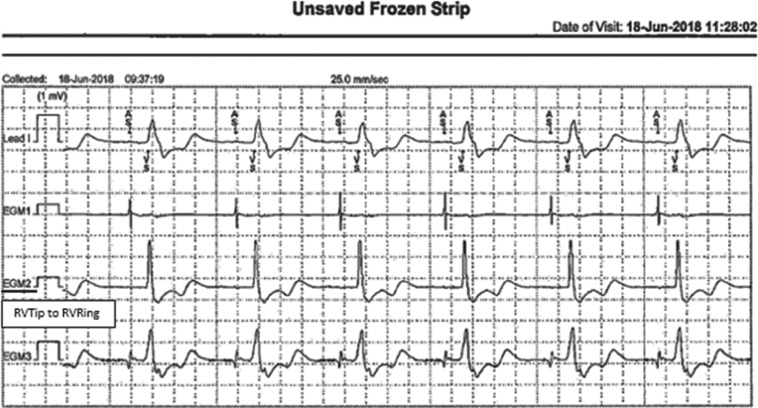
Intracardiac frozen strip with wide QRS pattern (loss of His-bundle capture).

**Figure 6:**
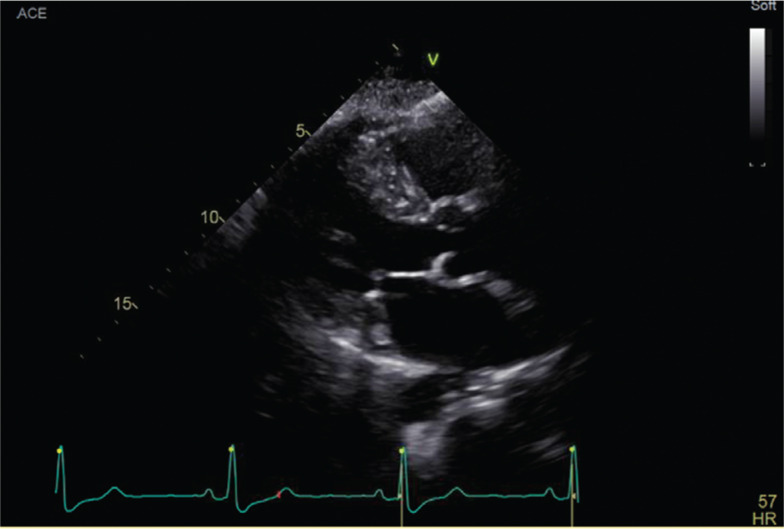
Echocardiogram after device implant.

In June 2018, RM showed an alert for “high right-ventricular (RV) thresholds” in the His position. The patient called the office and stated her chest pain had returned; the presenting intracardiac electrogram showed a wide QRS pattern **(Figure 5)**. An in-office interrogation revealed a loss of His-bundle capture secondary to a suboptimal output setting (elevated His-bundle threshold). The His-bundle lead output was programmed to 4.2 V, with the ECG showing QRS normalization and without the patient reporting further complaints of chest pain. The ventricular capture algorithm was programmed to “monitor” only. The patient continues to undergo semiannual in-office checkups scheduled alongside continued RM. In July 2019, an echocardiogram showed an improved LVEF of 62% with paradoxical septal wall motion improvement **(Figure 6)**.

## Discussion

Painful LBBB syndrome is an under-recognized condition that can occur in patients, irrespective of the presence of coronary disease.^1,2^ Few cases have been reported to date in the literature, and no specific treatments are available at this time.^3^ Case presentations of sudden chest pain with a LBBB appearance and chest pain resolution with the disappearance of LBBB aberrancy have been reported.^4^ Theories for this have been introduced, including cardiac mechanical dyssynchrony; clinical and ECG characteristic features have also been proposed in order to provide more guided and appropriate therapies, which have included β-blockers, exercise, and RV or biventricular pacing options.^4^ More novel technologies such as His-bundle pacing, which is a more physiologic and “natural” way to activate the conduction system, have been used with success in painful LBBB syndrome.^5^ Of note, the lack of a myocardial-evoked response to His-bundle capture can result in inaccurate and misleading capture management threshold measurements. Some have reported the unexpected loss of capture during atrial capture management in conjunction with certain biventricular pacemakers when the His-bundle lead is in the LV port and the RV channel is programmed to subthreshold outputs.^6^ The 2015 Heart Rhythm Society expert consensus statement on remote interrogation and monitoring^7^ advocates for the adoption of RM services as a standard of care in patients with CIEDs. While device clinic workflow challenges were recognized early on with RM implementation,^8^ RM systems offer greater follow-up efficiency, thus ensuring quicker clinical response can occur.

## Conclusion

**Figure 7:**
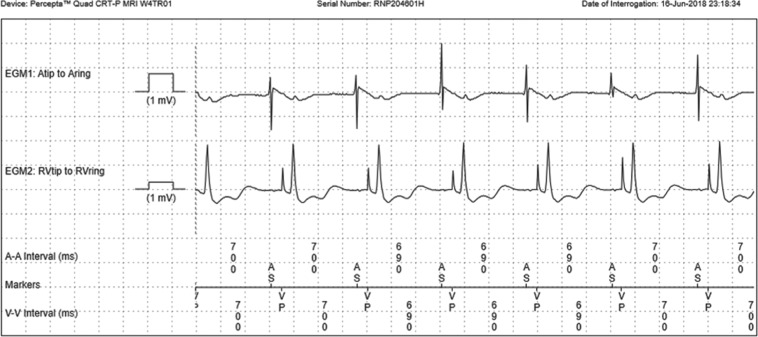
Intracardiac frozen strip with narrow QRS pattern (His-bundle capture).

In the presented case study, the patient was discharged after CRT-P implantation with RM, with connectivity confirmed during postprocedure follow-up. An alert for an elevated RV threshold at eight weeks after device implantation was followed by the return of her chest pain. His-bundle capture was confirmed when a manual transmission was requested **(Figure 7)**. However, the symptoms persisted, and an in-office interrogation performed 48 hours later revealed a loss of His-bundle capture **(Figure 5)**. Subsequently, an adjustment to regain capture was made, with the chest pain symptoms subsiding and resolving during the same office visit. The patient did not return again to the ER with a similar complaint. Her “connected” clinical care allowed for patient reassurance during the episode, with resultant savings in health care utilization, increased patient confidence in the technology used to maintain vigilant device surveillance, a quicker response to an actionable event, and timely delivery of patient care.

## References

[1] Virtanen KS, Heikkila J, Kala R, Siltanen P (1982). Chest pain and rate-dependent left bundle branch block in patients with normal coronary angiograms.. Chest..

[2] Glancy DL, Khuri B (2001). Chest pain and left bundle branch block.. Proc (Bayl Univ Med Cent)..

[3] Malozzi C, Wenzel G, Karumbaiah K (2014). Chest pain associate with rate-related left bundle branch block and cardiac memory mimicking ischemia.. J Cardiol Cases..

[4] Shvilkin A, Ellis ER, Gervino E (2016). Painful left bundle branch block syndrome: clinical and electrocardiographic feature and further directions for evaluation and treatment.. Heart Rhythm..

[5] Suryanarayana PG, Frankel DS, Marchlinski FE, Schaller RD (2018). Painful left bundle branch block syndrome treated successfully with permanent His bundle pacing.. HeartRhythm Case Rep..

[6] Padala SK, Ellenbogen KA, Koneru JN (2017). Intermittent loss of capture in a His bundle pacemaker: what is the cause?. HeartRhythm Case Rep..

[7] Slotwiner D, Varma N, Akar JG (2015). HRS Expert Consensus Statement on remote interrogation and monitoring for cardiovascular implantable electronic devices.. Heart Rhythm..

[8] Cronin EM, Ching EA, Varma N, Martin DO, Wilkoff BL, Lindsay BD (2012). Remote monitoring of cardiovascular devices: a time and activity analysis.. Heart Rhythm..

